# Felony disenfranchisement laws and racial inequities in women's self-rated health

**DOI:** 10.3389/fpubh.2025.1555227

**Published:** 2025-02-27

**Authors:** Anna K. Hing, Jé Judson, Marian Candil Escobar

**Affiliations:** ^1^Elson S. Floyd College of Medicine, Washington State University Spokane, Spokane, WA, United States; ^2^Division of Epidemiology and Community Health, University of Minnesota Twin Cities, St. Paul, MN, United States; ^3^Regenerative Agriculture Alliance, Northfield, MN, United States

**Keywords:** felony disenfranchisement, health disparities, state laws, structural racism, health equity

## Abstract

**Objectives:**

To determine if more strict state-level felony disenfranchisement laws, which are a form of structural racism, are associated with worse self-rated health, and if this association is stronger for Black women compared to white women.

**Methods:**

Using Behavioral Risk Factor Surveillance System (BRFSS) 2021, American Community Survey 2017–2021, and State Felony Disenfranchisement Laws in 2020 from the “Locked Out Report” by the Sentencing Project, we fit hierarchical linear models to estimate changes in self-rated health with state felony disenfranchisement laws for 185,833 Black and white women, stratified by race, in 49 states (excluding Florida).

**Results:**

We found a significant positive association between more restrictive disenfranchisement and worse self-rated health for Black women (*b* = 0.08, SE = 0.03, *p* < 0.01), but not white women, in the fully adjusted model.

**Conclusions:**

Stricter state-level felony disenfranchisement laws were associated with worse self-rated health for Black women but not white women suggesting that policies of disenfranchisement may exacerbate racial inequities in health.

## Introduction

In 2020, *5.2 million people*, or 2.3% of the voting age population, were denied their right to vote due to a felony conviction (1). The percentage of people disenfranchised varies by state's felony disenfranchisement laws, but in Alabama, Mississippi, and Tennessee, as much as 8% of the voting age population is prevented from voting. Voting, or conversely being prevented from voting, could be an important determinant of health (2).

Research suggests that 1 in 16 Black Americans of voting age are prevented from voting in the U.S., but as high as *1 in 7* are disenfranchised in Alabama, Kentucky, Florida, Mississippi, Tennessee, Virginia, and Wyoming (1). Nationally, 6.2%, or 2.5 million, Black voters cannot vote due to felony disenfranchisement laws. The disproportionate impact on Black voters is by design; felony disenfranchisement laws are grounded in white supremacy and have a legacy of targeting Black voters dating back to Reconstruction (3, 4) with disproportionate convictions of Black people continuing into today.

Voter disenfranchisement is a key mechanism through which racialized people are *symbolically* removed from society while incarceration is a key mechanism through which racialized people are *physically* removed from society. Thus, felony disenfranchisement laws exacerbate the marginalization of those with felony convictions by extending their punishment beyond incarceration into parole and probation, and even further into their futures. The strictness of felony disenfranchisement laws varies by state. For example, two states allow people incarcerated for a felony to vote even while imprisoned, while most place some restrictions on voting during incarceration, parole, probation, and beyond. Although there have been more recent efforts to loosen restrictions in a handful of states, these laws have remained relatively static over time.

Felony disenfranchisement has known racialized implications for health. Lukachko et al. (5) found it was associated with myocardial infarction for Black, but not white people. Similarly, Homan and Brown (6) found that higher levels of racialized disenfranchisement were associated with more depressive symptoms, functional limitations, and difficulty performing activities of daily living for Black, but not white, older adults. Thus, voting power and disenfranchisement may be an overlooked contributor to the persistent racialized inequities in women's health.

Inequities in health between Black and white women are well documented. Black women face higher rates of maternal mortality and morbidity, preterm birth, and infant mortality (7, 8), and have higher rates of cardiovascular disease (9), hypertension, lupus, (10) and other diseases compared to white women (11, 12). These disparities are not due to race, rather, they are caused by exposure to racism. Through the process of weathering, which suggests that due to repeated and cumulative exposure to racism and sexism, and subsequent social and economic disadvantage, Black women experience premature physical deterioration compared to white women (13). As felony disenfranchisement is a form of structural racism (3, 4, 6), it is possible that these policies contribute to worsened health for Black women through this process of weathering. While there is little recent data about Black women's disenfranchisement specifically, in 2022 nearly 1 million women were disenfranchised (14). Given that Black women are incarcerated at 1.6 times the rate of white women (15), there is likely a disproportionate impact on Black women voters. Therefore, as research continues to identify mass incarceration as a factor associated with health inequities for Black women (16–18), we conceptualize felony disenfranchisement laws as the next area for research in the continuum of the criminal legal process from arrest to incarceration, parole, probation, and beyond.

In this national study, we examine the impact of state-level felony disenfranchisement laws on self-rated health for Black and white women to understand how this specific type of structural racism works to exacerbate racialized health inequities. These felony disenfranchisement laws marginalize racialized groups, while preserving voting rights for people racialized as white. Thus, we would expect white women to not be impacted or to even be positively impacted by these laws, while we would expect Black women to have worse health. We hypothesize that more strict felony disenfranchisement laws will be associated with worse health outcomes, and that this association will be stronger for Black women.

## Materials and methods

### Population and data

We used individual-level data from the Behavioral Risk Factor Surveillance System (BRFSS) 2021, and included women who identified as white or Black in this study across all states, except Florida for which data were incomplete. This resulted in a sample of 185,833 Black and white female-identifying residents in 49 states. State-level covariate data came from the American Community Survey 2017–2021 data. Data on felony disenfranchisement laws (2020) was collected from the “Locked Out Report” by the Sentencing Project (1). Black and white women were chosen for this study because felony disenfranchisement laws especially impact Black people, are an extension of slavery and anti-Black racism in the US, and function to uphold white supremacy and benefit white people.

### Outcome

Self-rated health was used as the outcome for this study, measured as 1 “excellent” to 5 “poor.” Self-rated health has been used as a holistic indicator of health to capture physical, mental, and social health (19). Consistent with others (20–22), self-rated health was treated as a continuous variable because it provides results that are easily interpretable.

### Exposure

Across the United States, state-level felony disenfranchisement laws can be grouped into five categories based on their level of voting restrictiveness for persons with a felony conviction. These categorizations are: no restrictions; prison only restrictions; prison and parole restrictions; prison, parole, and probation restrictions; and beyond probation restrictions. This study created a dichotomous variable of less and more restrictive laws to categorize the various levels. “Less” referred to states that have no restrictions to voting for persons with felony convictions or only restrict voting for people with felonies while they are in prison. “More” referred to states that restricted voting during both prison and parole; prison, parole, and probation; or beyond probation. While theoretically it would have been more compelling to compare states with any restrictions to states with no restrictions, only two states fit into the no restrictions category (Maine and Vermont) and that grouping was too imbalanced for analyses. See Table 1 for how each state was grouped based on their laws in 2020.

**Table 1:** States by type of felony disenfranchisement laws 2020 (12).

**No restrictions**	**Prison only**	**Prison and parole**	**Prison, parole, and probation**	**Prison, parole, probation, and beyond**
Maine Vermont	Colorado Hawaii Illinois Indiana Maryland Massachusetts Michigan Montana Nevada New Hampshire New Jersey North Dakota Ohio Oregon Pennsylvania Rhode Island Utah	California Connecticut New York	Alaska Arkansas Georgia Idaho Kansas Louisiana Minnesota Missouri New Mexico North Carolina Oklahoma South Carolina South Dakota Texas Washington West Virginia Wisconsin	Alabama Arizona Delaware Iowa Kentucky Mississippi Nebraska Tennessee Virginia Wyoming

### Covariates

All models included state fixed effects with a random intercept to control for geographic differences. Additional state-level covariates were included due to their connection to both the exposure and outcome. Demographic and economic indicators were obtained from the American Community Survey data including the percent of the population that identified as Black and median income. Additional state data included party control of the state government (Republican, Democrat, or split), and former Jim Crow state (dichotomously coded) as these may be a determinant of the type of law passed and may influence other social factors shaping health for racialized people. Individual-level covariates from BRFSS included age (coded as 13 five-year increments from 18 years to 80 years and older), college education (dichotomous), unemployed (dichotomous), and any insurance (dichotomous).

### Statistical analysis

Descriptive analyses compared Black and white women living in states with less/more restrictions to states with high restrictions. We then fit hierarchical linear models to estimate changes in self-rated health with felony disenfranchisement laws because of the nested nature of individuals within states, and included a random intercept for state and random error. Three models were run starting with a simple bivariate analysis of felony disenfranchisement laws and Self-rated health. We then added state covariates in model 2, and individual level covariates were additionally added in model 3. As the goal of this analysis is to understand if and how structural racism differentially impacts the health of Black women compared to white women, all regression analyses were stratified by race. These analyses were conducted in StataMP, version (23). While BRFSS data can be weighted, we chose not to apply the weights for population estimates, consistent with prior research (24–26). As we are not trying to generalize about the overall prevalence of poor self-rated health in the country, it is not essential to have population estimates. When weights are applied, sampling variance, standard deviation, and standard errors increase, reducing accuracy. Rather, we are interested in estimating the effect of felony disenfranchisement laws on health, and as such, prioritized that accuracy over having a sample reflective of the US population. Additionally, we are already working with a reduced sample of Black and white women using only complete cases in BRFSS so this data is not representative of the general population.

### IRB

This study was exempt from IRB approval as no human subjects data were used, and all data were publicly available.

## Results

Of the 49 states included in this analysis, 19 were categorized as less restrictive felony disenfranchisement states while 30 were categorized as more restrictive (Table 2). Self-rated health varied between the two types of states, as well as for Black and white women. Black women in more restrictive felony disenfranchisement states had the worst average self-rated health compared to any other race-by-level of restrictiveness group (self-rated health = 2.8). Even in less restrictive states, Black women (self-rated health = 2.6) still fared worse, on average, than white women in any state (less restrictive self-rated health = 2.4, more restrictive self-rated health = 2.5). Women in more restrictive felony disenfranchisement states tended to be older and slightly less educated. Also of note is that 63% of more restrictive states were former Jim Crow states. More restrictive states also had a larger Black population, lower median income, and state governments were more Republican controlled.

**Table 2:** Means and percents for 2020 felony disenfranchisement laws, American Community Survey 2017–2021 and Behavioral Risk Factor Surveillance System 2021 (*N* = 50 states, 185,833 women).

**Level 1—Individual** ***N*** = **185,833**				
	**Black**		**White**	
	**Less restrictive felony disenfranchisement laws** ***N*** = **5,247**	**More restrictive felony disenfranchisement laws** ***N*** = **11,843**	**Less restrictive felony disenfranchisement laws** ***N*** = **59,992**	**More restrictive felony disenfranchisement laws** ***N*** = **108,751**
Self-rated health (range 1/excellent−5/poor)	2.6 (1.0)	2.8 (1.0)	2.4 (1.0)	2.5 (1.0)
Number of days of poor physical health in the past month	3.7 (7.9)	4.4 (8.7)	3.9 (8.3)	4.1 (8.5)
Mean age (5-year category ranging from 1 to 13)	6.9 (3.4)	7.3 (3.4)	8.0 (3.4)	8.1 (3.4)
College education or higher	40%	35%	46%	41%
Unemployed	8%	7%	4%	4%
Any insurance	97%	95%	97%	96%
**Level 2—State** ***N*** = **49**				
	**Black**		**White**	
	**Less restrictive felony disenfranchisement laws** ***N*** = **19**	**More restrictive felony disenfranchisement laws** ***N*** = **30**	**Less restrictive felony disenfranchisement laws** ***N*** = **19**	**More restrictive felony disenfranchisement laws** ***N*** = **30**
**Government trifecta**				
Democratic	47%	23%	47%	23%
Split	21%	27%	21%	27%
Republican	32%	50%	32%	50%
Former Jim Crow state	5%	63%	5%	63%
Percent black	17.5 (7.3)	19.6 (10.4)	8.1 (7.3)	10.8 (10.4)
Median income	78784.9 (10620.4)	64,762 (10317.6)	72929.6 (10620.4)	67623.7 (9429.12)

In the bivariate analysis regressing high felony disenfranchisement laws on self-rated health for Black women (Table 3A, Model 1), there was a significant positive association between more restrictive disenfranchisement and worse self-rated health (*b* = 0.19, SE = 0.04, *p* < 0.001) compared to less restrictive states. This relationship persists when state-level covariates are added, though it is slightly attenuated (Model 2, *b* = 0.08, SE = 0.03, *p* < 0.01), and remains significant even when individual-level controls are added (Model 3, b = 0.06, SE = 0.03, *p* < 0.05). Thus, in the fully adjusted model, we see a 0.06 increase in worse self-rated health for Black women who live in more restrictive states compared to Black women living in less restrictive states. For white women (Table 3B), we observe that while there is also an initial positive association between living in a more restrictive state and worse self-rated health (Model 1, *b* = 0.11, SE = 0.03, *p* < 0.001), the significance of this relationship disappears as state and individual-level controls are added in subsequent models, with no association in the fully adjusted model (Model 3, *b* = 0.00, SE = 0.02, *p* > 0.05). In conclusion, we see that more restrictive felony disenfranchisement laws are significantly associated with worse self-rated health for Black women, but not white women, in fully adjusted models.

**Table 3A:** Multilevel regression analysis of felony disenfranchisement laws and self-rated health for Black women: 2020 felony disenfranchisement laws, American Community Survey 2017–2021 and Behavioral Risk Factor Surveillance System 2021 (*N* = 49 states, 17,090 individuals).

	**Model 1**	**Model 2**	**Model 3**
**Variables**	β **(SE)**	β **(SE)**	β **(SE)**
**Felony disenfranchisement law**			
Less restrictive (No disenfranchisement or prison only)	Ref	Ref	Ref
More restrictive (Parole, probation, or longer)	0.19 (0.04)^***^	0.08 (0.03)^**^	0.06 (0.03)^*^
**Party control**			
Democrat	Ref	Ref	Ref
Split		0.08 (0.04)^*^	0.03 (0.03)
Republican		0.10 (0.04)^**^	0.06 (0.03)
Former Jim Crow state		−0.02 (0.04)	−0.03 (0.03)
Percent Black		0.00 (0.00)	−0.00 (0.00)
Median income		0.00 (0.00)^***^	−0.00 (0.00)^***^
Percent with any insurance coverage		0.07 (0.04)	−0.03 (0.04)
Age			0.08 (0.00)
College educated			−0.35 (0.02)^***^
Unemployed			0.07 (0.03)^*^
Constant	2.61 (0.04)^***^	3.05 (0.11)^***^	2.65 (0.10)^***^
−2 Log Likelihood	−24918.40	−24896.72	−24085.86

^*^p < 0.05; ^**^p < 0.01; ^***^p < 0.001.

**Table 3B:** Multilevel regression analysis of felony disenfranchisement laws and self-rated health for white women: 2020 felony disenfranchisement laws, American Community Survey 2017–2021 and Behavioral Risk Factor Surveillance System 2021 (*N* = 49 states, 168,743 individuals).

	**Model 1**	**Model 2**	**Model 3**
**Variables**	β **(SE)**	β **(SE)**	β **(SE)**
**Felony disenfranchisement law**			
Less restrictive (No disenfranchisement or prison only)	Ref	Ref	Ref
More restrictive (Parole, probation, or longer)	0.11 (0.03)^***^	0.01 (0.02)	0.00 (0.02)
**Party control**			
Democrat	Ref	Ref	Ref
Split		−0.01 (0.02)	−0.01 (0.03)
Republican		0.03 (0.03)	−0.00 (0.02)
Former Jim Crow state		0.07 (0.03)^*^	0.06 (0.03)^*^
Percent Black		0.00 (0.00)	0.00 (0.00)
Median income		0.00 (0.00)^***^	0.00 (0.00)^***^
Percent with any insurance coverage		0.10 (0.01)^***^	−0.08 (0.01)^***^
Age			0.04 (0.00)^***^
College educated			−0.41 (0.00)^***^
Unemployed			0.26 (0.01)^***^
Constant	2.41 (0.03)	2.92 (0.09)^***^	2.69 (0.08)^***^
−2 Log Likelihood	−243182.33	−243131.82	−237659.97

^*^p < 0.05; ^**^p < 0.01; ^***^p < 0.001.

### Limitations

This study is the first to examine how felony disenfranchisement laws are associated with self-rated health, yet it does have some limitations. First, Florida was excluded from analyses due to data limitations. Florida is a state with some of the harshest felony disenfranchisement laws historically and has experienced dynamic changes in its laws in the past 6 years, showing how even when felony voting rights are restored other means such as financial obligations can be leveraged to extend disenfranchisement. Second, felony disenfranchisement was coded dichotomously because of underrepresentation in some categories, which may have masked a more nuanced understanding of the impact of each category of law. It is also possible that a lag time for the effect of laws exists and states that saw recent changes in their laws may be miscategorized. For example, in states where laws become less restrictive, people with a history of a felony may be reluctant to vote due to fear that it is still illegal and they could face additional punishment (27).

## Discussion

Stricter state-level felony disenfranchisement laws are associated with lower self-rated health for Black women, but not white women. This is consistent with a racism conscious framework, as informed by Public Health Critical Race Praxis (28), in which we consider that racism disproportionately harms racialized people and works to uphold white supremacy, not just socially or economically, but also physically. The weathering (13) that Black women experience in the face of structural racism contributes to an increased allostatic load that may not manifest as just one disease, but as poor health overall. One study found that felony disenfranchisement rates were associated with greater sexually transmitted disease prevalence in women, but did not stratify results by racialized group (29). The observed 0.06 increase in poor self-rated health score for Black women translates to a 12% decline in health, and, if we consider felony disenfranchisement as one isolated form of structural racism, we can begin to see how the totality of ways that structural racism influences health can compound upon one another to have an even greater impact on the health of racialized communities.

Voter suppression laws and felony disenfranchisement laws in particular are one form of structural racism (3, 4, 6). Structural racism is “the totality of how society is organized to privilege white communities at the expense of non-white racialized communities” (30). This structural privilege is built upon the ideology of white supremacy and functions through the interconnections among the different domains and institutions that maintain and reinforce this structure, including the carceral and political domains. Structural racism serves to concentrate power along racialized lines (2–4).

Voter disenfranchisement laws of today developed from a lineage of racist laws and policies (3). In 1965, the Voting Rights Act was passed which sought to address the discrimination experienced at the polls, especially by Black voters. The VRA had almost immediate consequences for Black voters, resulting in increased Black voter turnout, yet felony disenfranchisement laws have persisted in disproportionately disqualifying Black voters. Rushovich and colleagues found that the VRA was associated with reductions in Black infant mortality rates in former Jim Crow States (31). Thus, we can see clearly that protections for voting rights are one step to reverse the impacts of racist policies and improve health for racialized people. Their study, which considered birth outcomes, is instructive in considering how racist policies influence birthing people specifically, and the intergenerational consequences of such policies (31).

Further, this study illustrates the impact of felony disenfranchisement laws on the health of people who likely are not the ones being directly disenfranchised. We cannot identify people formerly incarcerated for a felony conviction in this analysis, but a majority of people in this sample likely are experiencing the indirect effect of these laws on their health in statistically significant ways. Approximately 4.7 million of the 6.1 million disenfranchised in 2017 were living in communities on release, not in prison (3). The expansion of the vote to people convicted for a felony may help to move the needle toward health equity.

Understanding the link between felony disenfranchisement, adverse health policies and conditions, and health disparities provides added evidence to inform policy change for racial justice in health. While prior papers have linked political disenfranchisement (2) and felony disenfranchisement disparities to health disparities (6, 32), no empirical analyses have tested the connection between felony disenfranchisement laws and health for Black and white women. The examination of felony disenfranchisement policies, as opposed to racial ratios of the number of people disenfranchised, is important because it names the law as the embodiment of structural racism rather than looking at the outcome of the law as a proxy for the form of structural racism. Identifying laws also provides a clear target for intervention, which can be obfuscated when looking at the outcomes of such laws. Further, the study of felony disenfranchisement laws, as compared to other forms of voter suppression, is important because these laws are one way in which the effects of racism in the carceral and policing systems are expanded. The interinstitutional connections linking two different domains of racism illustrate how the potential reach of racism is magnified and upheld (30). Future research should move to examine not only ecological, national studies using specific policies, but also investigate how health changes when felony disenfranchisement laws are altered, such as in Minnesota or Florida.

Beyond health consequences, these laws influence the outcomes of elections, and, by extension, the landscape of policies that shape known social determinants of health, including housing, social welfare, and Medicaid expansion. In 2016, the difference in the popular vote between the two Presidential candidates was 2.87 million votes. If we consider that 5.2 million people were disenfranchised at the time we can see the potential impact these missing votes can have on improving people's everyday lives. By examining the association for non-incarcerated individuals and those without felony convictions, these findings suggest an indirect influence of racist laws on the Black friends and family of those who are disenfranchised, perhaps via stigma, unfair treatment, and diluted political power (6). The entire Black voting bloc is weakened given the effectual silencing of 2.5 million Black voices, making it difficult for Black voters to elect officials and pass relevant policies that may reduce or eliminate racial inequities in health.

Several states have engaged in recent policy changes regarding voting rights for those with felony convictions. In 2023, Minnesota voted to reduce voting restrictions so that people with a felony conviction can vote upon release, restoring voting rights to an estimated 55,000 people (33). In Arizona as of 2021, voting rights are restored only after all terms of the sentence are completed, including parole and probation, and paid all restitution, which disenfranchises about 200,000 people, of which a majority are Black or Latino (34). If the franchise were expanded, those 200,000 could be eligible to vote. Even reducing permanent disenfranchisement, such as occurred in Iowa in 2020 through an executive order can re-enfranchise tens of thousands of people (35). The expansion of voting rights, even moving from permanent disenfranchisement to disenfranchisement until sentences are complete, would expand the vote to thousands of people, while allowing anyone to vote, as is done in Maine and Vermont, would re-enfranchise millions. These millions, mostly from racialized groups, would be given back their political voice and agency to shape laws and policies that support their health and the health of their communities. Thus, felony disenfranchisement is both a carceral and political form of disenfranchisement, and its impact is both on the health of those who are incarcerated and, as we found, even the Black female population, generally. At the federal level, the John Lewis Voting Rights Advancement Act is a contemporary approach to address the *Shelby County v. Holder* decision which reversed many of the voting protections against racial discrimination established by the VRA, expanding its reach beyond felony disenfranchisement laws to remedy discrimination in voting laws more broadly. However, even at the state-level, restrictive felony disenfranchisement laws can be changed.

## Conclusion

We must consider health in all policies, especially voting policies (2). These analyses show that state-level felony disenfranchisement laws are associated with worse self-rated health for Black women but not white women, demonstrating that these laws may contribute to maintaining racial health inequities. If we consider all other ways in which structural racism is woven into policies and institutions and daily interactions for racialized people, we can begin to understand the cumulative impact of structural racism and why it appears to be so intractable. To move toward health equity, we must expand people's access to power (36) by bringing them back into society, not banning them further. This is not just good for them, but good for everyone. Policymakers and researchers should consider not only health in all policies, but if and how racism is present in all policies in order to move toward health equity.

## Data Availability

The original contributions presented in the study are included in the article, further inquiries can be directed to the corresponding author.
